# Correction to: Upregulating CXCR4 in Human Fetal Mesenchymal Stem Cells Enhances Engraftment and Bone Mechanics in a Mouse Model of Osteogenesis Imperfecta

**DOI:** 10.1093/stcltm/szaf027

**Published:** 2025-06-14

This is a correction to: Gemma N. Jones, Dafni Moschidou, Kenneth Lay, Hassan Abdulrazzak, Maximilien Vanleene, Sandra J. Shefelbine, Julia Polak, Paolo de Coppi, Nicholas M. Fisk, Pascale V. Guillot, Upregulating CXCR4 in Human Fetal Mesenchymal Stem Cells Enhances Engraftment and Bone Mechanics in a Mouse Model of Osteogenesis Imperfecta, *Stem Cells Translational Medicine*, Volume 1, Issue 1, January 2012, Pages 70–78, https://doi.org/10.5966/sctm.2011-0007

In April 2024, the authors alerted the Editors to concerns regarding an overlapping field of view in Figure 4B, which concerns have also been raised via PubPeer (https://pubpeer.com/publications/7FA1AEAFE123E7BB26BBA949F8F988#1).

As part of the inquiry into Figure 4B, the authors checked the raw data in detail and confirmed that this was due to a clerical error in image presentation and does not modify the raw results or their interpretation of the results. A revised version of Figure 4B from the authors is provided below.


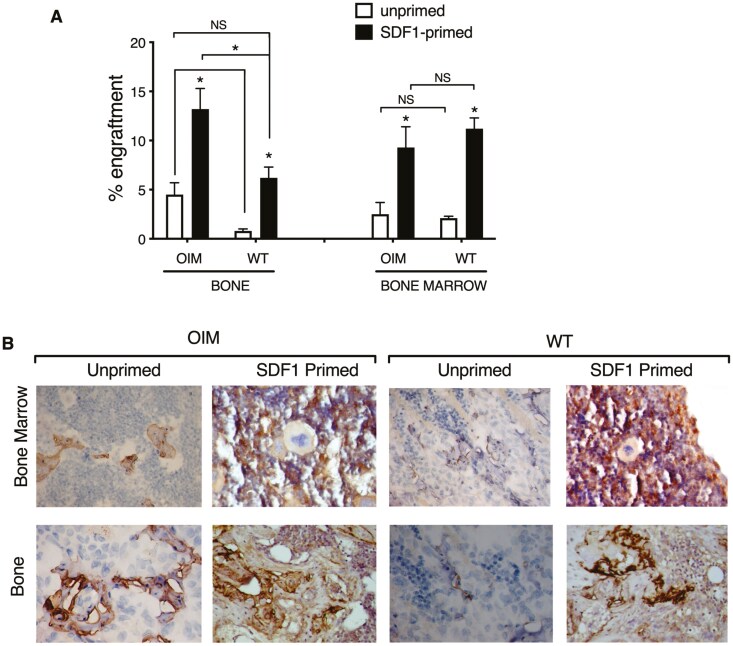


The Editors agree with the authors’ assertions that the findings of the paper are unaffected by these errors. The correction has been made only in this correction notice so as to preserve the published Version of Record.

